# Single‐cell transcriptomics and metabolomic analysis reveal adenosine‐derived metabolites over‐representation in pseudohypoxic neuroendocrine tumours

**DOI:** 10.1002/ctm2.70159

**Published:** 2025-02-04

**Authors:** Yuval Kahan Yossef, Liav Sela Peremen, Alona Telerman, Gil Goldinger, Sergey Malitsky, Maxim Itkin, Reut Halperin, Naama Peshes Yaloz, Amit Tirosh

**Affiliations:** ^1^ Tel Aviv University Faculty of Medicine Tel‐Aviv Israel; ^2^ ENTIRE ‐ Endocrine Neoplasia Translational Research Center Research Center for Endocrinology Diabetes and Metabolism Ramat Gan Israel; ^3^ Institute of Pathology Chaim Sheba Medical Center Tel HaShomer Israel; ^4^ Life Science Core Facilities Weizmann Institute of Science Rehovot Israel

1

Dear Editor,

Von Hippel−Lindau protein (pVHL) is a critical factor in the cellular oxygen sensing apparatus. pVHL‐deficient tumours are characterized by a pseudohypoxic state and a consequent metabolic shift towards anaerobic metabolism. Based on unbiased metabolic analysis, supported by single‐cell transcriptomics analysis, we report a potential tumorigenic role of adenosine in pVHL‐deficient pancreatic neuroendocrine tumors (vPNET).

VHL disease, caused by germline DNA pathogenic variants (PVs) in the *VHL* gene,^1^ is associated with predisposition for pancreatic neuroendocrine tumours (PNETs); hemangioblastoma(s) of the cerebellum, spine and retina; pheochromocytoma and paraganglioma, and renal cell carcinoma of clear‐cell type.^1^

The pVHL serves as the recognition unit of the ubiquitin system and identifies hypoxia‐inducible factor 1α (HIF1α) to promote its degradation.^2^, ^3^ pVHL‐deficient states lead to HIF1α accumulation and pseudohypoxia,^4^, ^5^ which promotes tumorigenesis and tumour progression and prompts a metabolic shift from oxidative pyruvate breakdown towards anaerobic glucose utilization.^2^, ^6^, ^7^

Somatic *VHL* PVs are exceedingly rare in sporadic PNET (sPNET).^8^ Hence, we hypothesized that *VHL* PV alone is insufficient for developing vPNET, and metabolic changes drive tumorigenesis. To elucidate this, we conducted tumour metabolomic profiling, single‐cell transcriptomic studies and tissue immunohistochemical characterization of vPNET and sPNET (please see full methods in the Supplementary Material).

The current work initiated with an unbiased metabolomic analysis to investigate the metabolic environment in patient‐derived tissue samples of vPNET and sPNET. Our analysis led to the putative identification of 217 polar metabolites (Supplementary Material) that demonstrated distinct metabolomic signatures and separation of vPNET versus sPNET (Figure 1A). To identify the metabolites that contributed most to the distinction between the groups, we performed a Variable Importance in Projection analysis, in which adenosine monophosphate (AMP) was identified as a dominant metabolite (Figure 1B). As shown in the volcano plot (Figure 1C) and heatmap (Figure 1D), vPNET had a higher representation of AMP as compared with sPNET.

**FIGURE 1:**
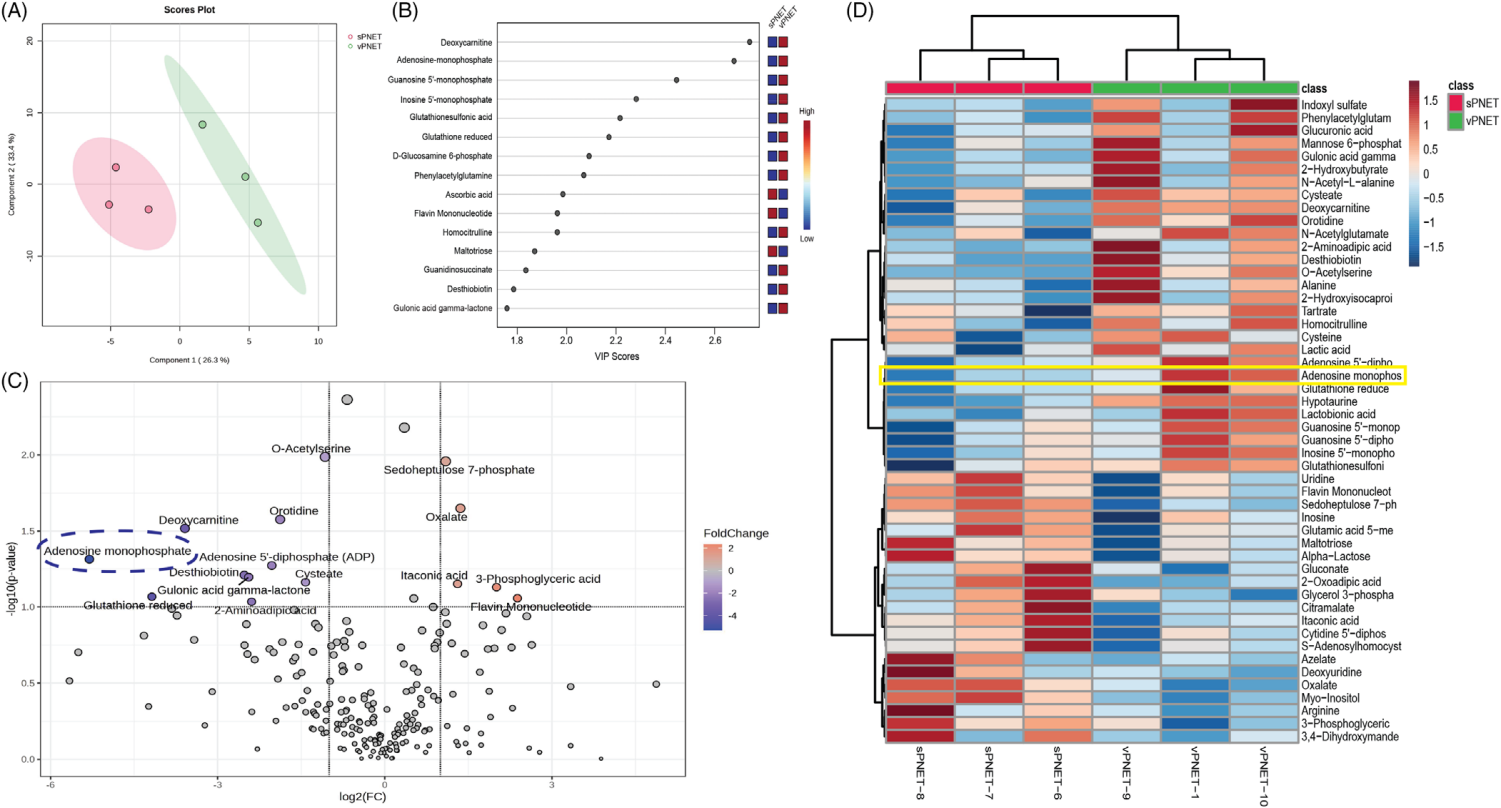
Metabolomic analysis of vPNET versus sPNET samples. Partial Least Squares Discriminant Analysis (PLS‐DA) plot demonstrates the clear separation between vPNET and sPNET samples (A), Variable Importance in Projection (VIP) plot highlights key metabolites influencing group differentiation, with adenosine monophosphate (AMP) identified as a major contributor (B), Volcano Plot displays significantly different metabolites, with those elevated in vPNET on the left and those in sPNET on the right. AMP is marked by a dashed circle, indicating its higher abundance in vPNET (C), and heatmap with an unsupervised hierarchical clustering, revealing unique metabolic signatures for vPNET (right) and sPNET (left), with AMP significantly higher in vPNET (yellow frame, D).

Other metabolites that were significantly differentially represented between the groups were less likely to be related to PNET tumorigenesis based on the literature review. To independently validate the metabolomics analysis findings, we performed an unbiased snRNA seq analysis. Single‐nucleus RNA sequencing was chosen, as it allows single‐cell transcriptomic analysis of frozen samples.

In the snRNA sequencing analysis, 25 982 high‐quality cells from two vPNET and five sPNET were identified and analysed. Using canonical correlation analysis integration across all samples, we identified eight cell types: acinar cells, neuroendocrine (NE) cells, including α, β and indeterminate, ductal cells, stellate cells, endothelial cells and immune cells. Figure 2A shows the UMAP of the various cells in the entire cohort, the markers defining each group are detailed in Figure 2B, and a UMAP of each sample in Figure 2C. Copy number alteration analysis demonstrates the accurate selection of NE cells based on their identification as the malignant component (Figure 2D,E). Consolidated copy number alteration analysis (Figure 2F) shows losses in chromosomes 3 and 11, and gains in chromosomes 7 and 13 in vPNET and sPNET. However, sPNET showed CN gains in chromosomes 4, 5, 17, 19 and 20, in contrast to losses in chromosomes 4 and 5 in vPNET. The cell representation in each sample (Figure 2E) demonstrates the identification of malignant NE cells and the relatively lower abundance of immune cells in sPNET versus vPNET.

**FIGURE 2:**
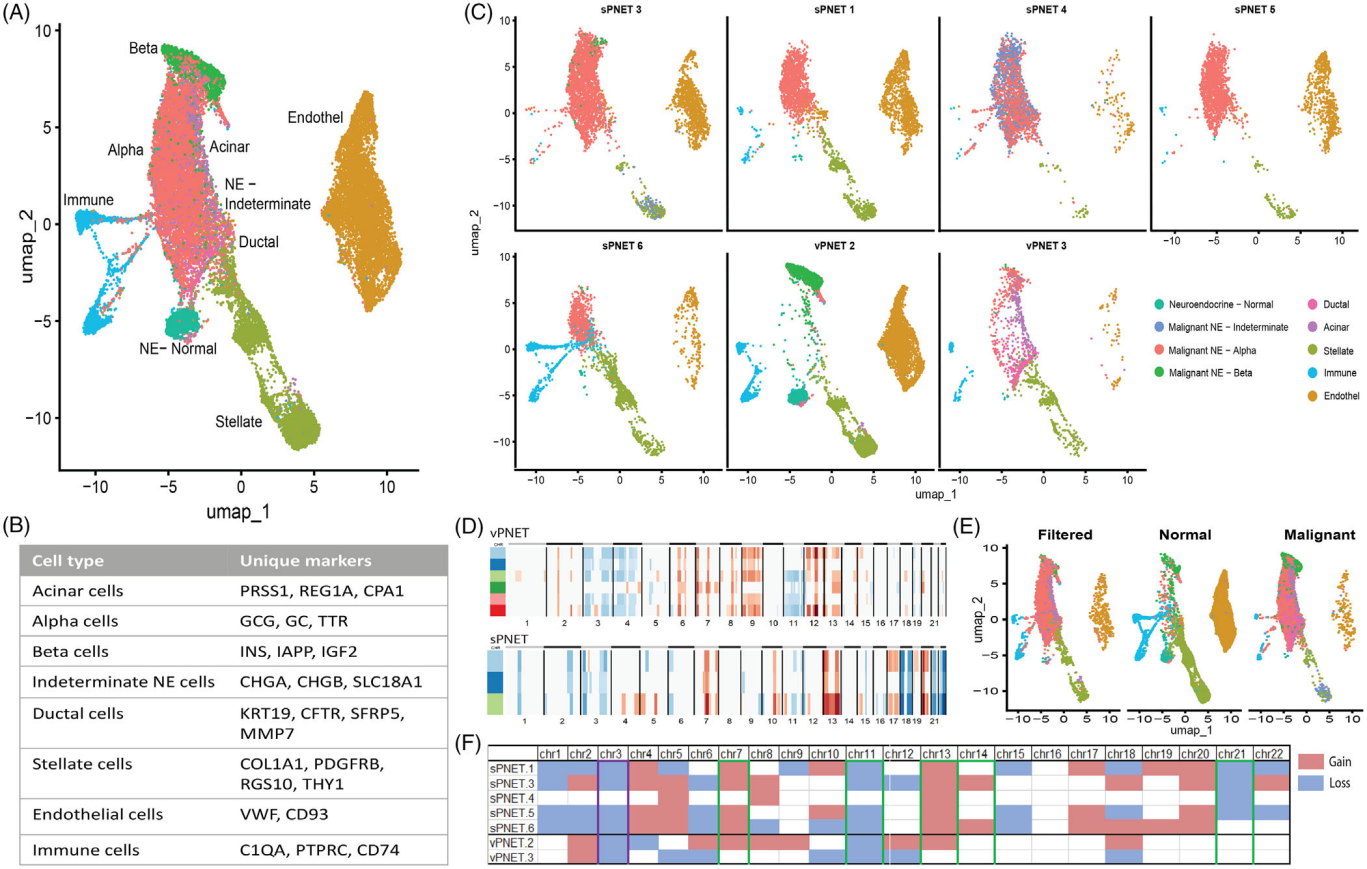
Single‐nucleus RNA sequencing (snRNA‐seq analysis) was used to identify cell populations in sPNET and vPNET samples. Uniform Manifold Approximation and Projection (UMAP) plot displays the various cell types identified across all samples (A), based on gene expression of cell‐specific biomarkers (B) and in each sample separately (C). Copy Number (CN) Variation analysis was conducted in vPNET and sPNET (upper and lower panel, respectively), to identify malignant and normal cells (D). Cells with CN alterations were identified as malignant, as shown in UMAP plots of the tumoral, normal and filtered‐out cells (E). CN alterations were summarized and compared between vPNET and sPNET samples (F).

Differential gene expression between vPNET and sPNET (Figure 3A) demonstrated upregulation of acinar cell markers (*PRSS1* and *PRSS2*) and β cell markers (*INS*) in vPNET as well as neuroendocrine markers (*CHGA*, *CHGB* and *SLC18A1*). In sPNET, we found relative upregulation of *ARX* and *TTR*, which are α cell markers, and *TNS3*, *MAPK4* and *MAPK10* associated with tumour development. In pathway analysis, based on these data, we observed enriched expression of genes related to hypoxia, glycolysis, apoptosis and the PI3K‐AKT‐MTOR pathways in vPNET versus sPNET (Figure 3B), in line with our findings in the metabolomic analysis. Metabolism‐pathways‐based enrichment analysis strengthened these results, identifying the enrichment of glycolysis, purine metabolism (the precursor of adenosine), energy metabolism and amino acid metabolism pathways (Figure 3C).

**FIGURE 3:**
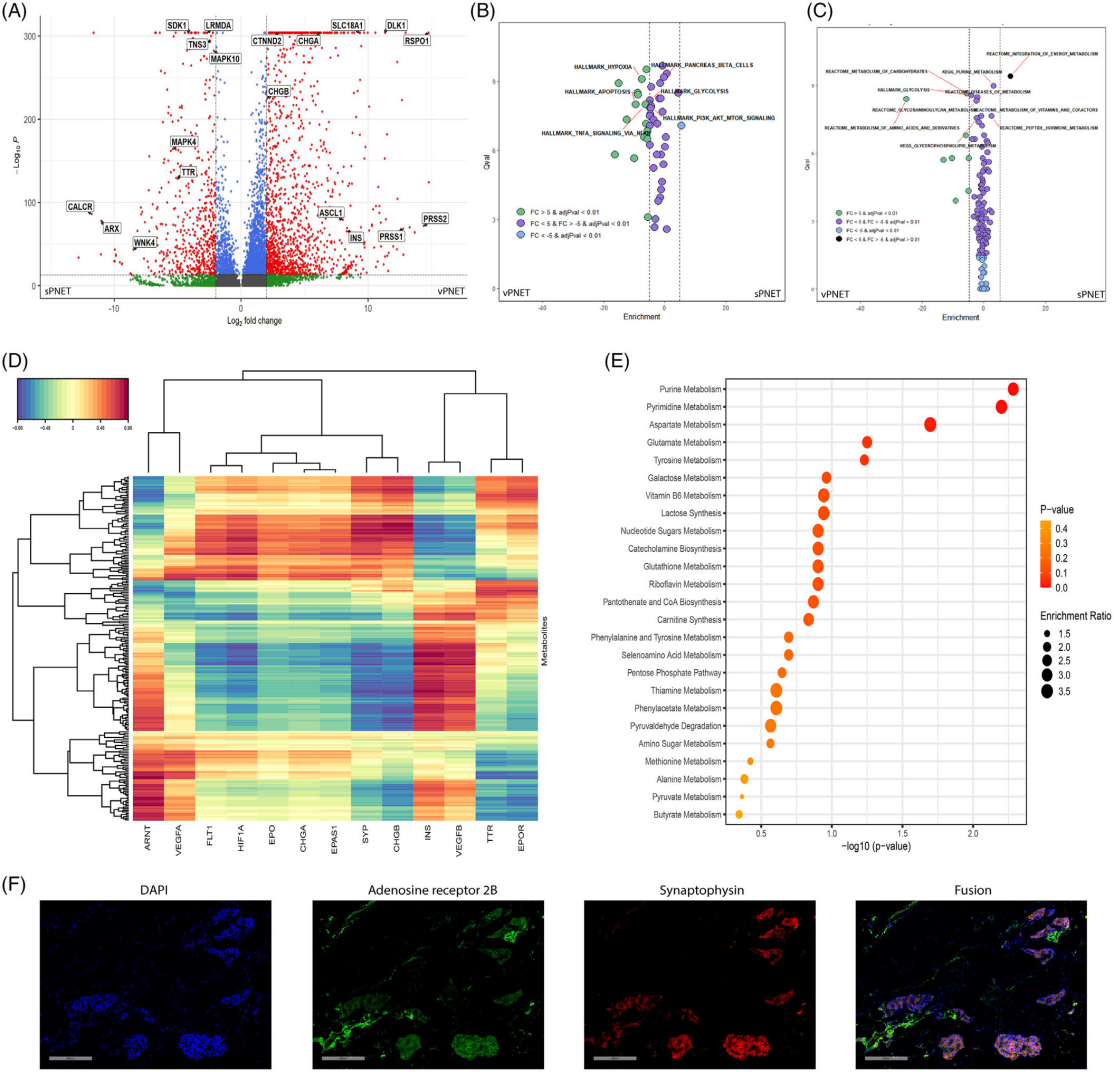
Pathway analysis and multi‐omics analysis of tumoral cells. Volcano plot displays the differential gene expression analysis of malignant cells in vPNET versus sPNET (A). Single‐cell pathway analysis comparing vPNET (left) to sPNET samples (right) based on Hallmark database (B), and on metabolic pathways from various databases (C). Integrated snRNA‐seq and metabolomics analysis, provided as a multi‐omics heatmap, depicting pseudohypoxia‐related genes across all cells from both vPNET and sPNET samples (D), and pathway enrichment, analysing the top 50 most variably detected metabolites from the multi‐omics data revealed purine metabolism as the most significantly enriched pathway (E). Immunofluorescence staining demonstrating expression of adenosine receptors in vPNET: neuroendocrine cells, identified by synaptophysin (red), adenosine receptor 2B (green) and DAPI staining (blue) to indicate nuclei, co‐localized in the merged image (F).

In multi‐omics analysis, *INS* (encoding insulin) expression, a beta cell marker, correlated with expression of the hypoxia‐related *VEGFA*, *VEGFB* and *ARNT* (Figure 3D), and examination of the 50 most variably represented metabolites, demonstrated purine metabolism, from which adenosine derives, as the most prominently enriched pathway (Figure 3E). Finally, we co‐localized protein expression of synaptophysin (NE cell marker) and adenosine receptor 2B^9^ on NE tumour cells in PNET (Figure 3F).

To study the tumoral evolution of NE cells, we performed cell trajectory and pseudo‐time analysis. In sPNET samples, the identified NE cell populations included normal (non‐malignant) NE cells and malignant cells that were sub‐grouped based on key gene expression into differentiated NE cells (expressing α‐ or β‐cell gene markers) or indeterminate, which did not express these genes. The trajectory analysis in sPNET indicated that normal NE cells were located on the early pseudo‐time, followed by indeterminate and differentiated NE cells (Figure 4A). In contrast, vPNET samples exhibited a different pseudo‐time evolution, with evolution involving normal NE cells and differentiated malignant NE cells, with a negligible representation of indeterminate cells (Figure 4B).

**FIGURE 4:**
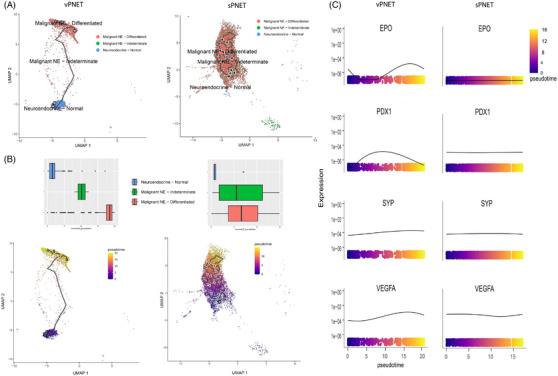
Cell trajectory and pseudo‐time analysis. Uniform Manifold Approximation and Projection (UMAP) visualization shows selected pseudo‐time points in neuroendocrine (NE) cells subgroups (A), and a bar‐plot showing the development of the different NE cells along the pseudo‐time axis (B). UMAP of NE cells coloured according to their pseudo‐time in vPNET (left) and sPNET (right) samples (C). Panel compares the expression patterns of key genes (e.g. *EPO*, *PDX1*, *SYP* and *VEGFA*) along the pseudo‐time trajectory in vPNET (left) and sPNET (right) samples (D).

Additionally, we analysed relevant genes and their expression along the pseudo‐time axis. We observed notable differences in gene expression patterns between sPNET and vPNET samples in the pseudo‐time‐dependent expression of hypoxia‐related genes (Figure 4C). While tumoral cell evolution was associated with increased expression of *EPO* and *VEFGA*, both directly upregulated by HIF, typical for pVHL‐deficient tumours, sPNET demonstrated stable expression of both genes.

The current analysis is limited by the small number of samples analysed. In addition, this is a descriptive study, which cannot determine the causality and dependency of pseudohypoxia, adenosine metabolite generation and mTOR pathway activation. For determining such causality, either in vitro studies or in vivo studies are required.

In conclusion, our data suggest that adenosine and purine derivatives are over‐represented in vPNET compared with sPNET, and possibly activate the mTOR pathway via adenosine receptors. Further in vitro studies are required to validate these results and the potential targetability of mTOR in vPNET.

## AUTHOR CONTRIBUTIONS

**Yuval Kahan Yossef**: Formal analysis, investigation, writing—original draft. **Liav Sela Peremen**: Investigation. **Alona Telerman**: Validation, supervision. **Gil Goldinger**: Validation. **Sergey Malitsky**: Writing—review and editing. **Maxim Itkin**: Investigation, writing—review and editing. **Reut Halperin**: Investigation, writing—review and editing. **Naama Peshes Yaloz**: Methodology, writing—review and editing, project administration. **Amit Tirosh**: Conceptualization, funding acquisition, supervision, writing—review and editing.

## CONFLICT OF INTEREST STATEMENT

The authors declare that they have no known competing financial interests or personal relationships that could have appeared to influence the work reported in this paper.

## ETHICAL APPROVAL STATEMENT

The study was approved by the Sheba MC Helsinki Committee (5674‐18‐SMC).

## Supporting information



Supporting Information

Supporting Information

Supporting Information

Supporting Information

## Data Availability

Data and full analysis script will be shared upon reasonable request from the corresponding author.

## References

[1] Lonser RR , Glenn GM , Walther M , et al. von Hippel−Lindau disease. Lancet. 2003;361(9374):2059‐2067. doi:10.1016/S0140-6736(03)13643-4 12814730

[2] Buckley M , Terwagne C , Ganner A , et al. Saturation genome editing maps the functional spectrum of pathogenic VHL alleles. Nat Genet. 2024;56(7):1446‐1455. doi:10.1038/s41588-024-01800-z 38969834 PMC11250436

[3] Jaakkola P , Mole DR , Tian YM , et al. Targeting of HIF‐alpha to the von Hippel−Lindau ubiquitylation complex by O2‐regulated prolyl hydroxylation. Science. 2001;292(5516):468‐472. doi:10.1126/science.1059796 11292861

[4] Maxwell PH , Wiesener MS , Chang GW , et al. The tumour suppressor protein VHL targets hypoxia‐inducible factors for oxygen‐dependent proteolysis. Nature. 1999;399(6733):271‐275. doi:10.1038/20459 10353251

[5] Wicks EE , Semenza GL . Hypoxia‐inducible factors: cancer progression and clinical translation. J Clin Invest. 2022;132(11):e159839. doi:10.1172/JCI159839 35642641 PMC9151701

[6] Kierans SJ , Taylor CT . Regulation of glycolysis by the hypoxia‐inducible factor (HIF): implications for cellular physiology. J Physiol. 2021;599(1):23‐37. doi:10.1113/JP280572 33006160

[7] Warburg O , Wind F , Negelein E . The metabolism of tumors in the human body. J Gen Physiol. 1927;8(6):519‐530. http://www.ncbi.nlm.nih.gov/pubmed/19872213 19872213 10.1085/jgp.8.6.519 PMC2140820

[8] Scarpa A , Chang DK , Nones K , et al. Whole‐genome landscape of pancreatic neuroendocrine tumours. Nature. 2017;543(7643):65‐71. doi:10.1038/nature21063 28199314

[9] Pacini ESA , Satori NA , Jackson EK , Godinho RO . Extracellular cAMP‐adenosine pathway signaling: a potential therapeutic target in chronic inflammatory airway diseases. Front Immunol. 2022;13:866097. doi:10.3389/fimmu.2022.866097 35479074 PMC9038211

