# Supplementary material for: Single‐cell transcriptomics and metabolomic analysis reveal adenosine‐derived metabolites over‐representation in pseudohypoxic neuroendocrine tumours
Source: Clin Transl Med. 2025 Feb 4;15(2):e70159. doi: 10.1002/ctm2.70159 (PMC11791754; doi:10.1002/ctm2.70159)
Supplement: Supplementary file 1 — Supporting Information [file CTM2-15-e70159-s002.docx]

**Supplementary data**

*Materials and Methods*

*The* data generated in this study are available upon request from the corresponding author.

This study was approved by the Sheba Medical Center Institutional Review Board (SMC-18-5674). All tissue samples were collected after obtaining written informed consent. Clinical data of samples used for the various analyses is detailed in **Supplementary Table 2**.

*Metabolomics*

This analysis is based on the initial metabolomic analysis of three vPNET samples and five sPNET samples. We performed an unbiased metabolomic analysis that was validated on an independent cohort of three vPNET and three sporadic PNET (sPNET) samples, which are presented here. The samples were processed for the quantification of polar metabolites.

*Metabolites extraction*

Extraction and analysis of polar metabolites were performed as previously described in Malitsky *et al*. (2016)^1^ with some modifications. Briefly, the tumors were ground to powder using metal balls, and ~15 mg of dry material was mixed with 1 mL of a pre-cooled (−20˚C) homogenous methanol:methyl-tert-butyl-ether (MTBE) 1:3 (v/v) mixture. The tubes were vortexed and then sonicated for 30 min in ice-cold sonication bath (taken for a brief vortex every 10 min). Then, double deinonized water (DDW): methanol (3:1, v/v) solution (0.5 mL) containing internal following standards: C13 and N15 labeled amino acids standard mix (1:1500, 767964, Sigma, St Louis, MO, USA) was added to the tubes followed by centrifugation. The upper, organic phase was discarded. The lower, polar phase was re-extracted as described above, with 0.5 mL of MTBE. The polar phase was lyophilized and stored at −80˚C until analysis.

*Liquid chromatography-mass spectrometry (LC-MS) polar metabolite detection*

For metabolic profiling of the polar phase samples, the lyophilized pellets were dissolved using 100 µl DDW-methanol (1:1), centrifuged twice (20800 rcf, 10 min) to remove possible precipitants, and were injected into LC-MS system.

Analysis was performed using an Acquity I class UPLC System combined with a mass spectrometer (Thermo Exactive Plus Orbitrap) operated in a negative ionization mode. The LC separation was done using the SeQuant Zic-pHilic (150 mm × 2.1 mm) with the SeQuant guard column (20 mm × 2.1 mm) (Merck). The Mobile Phase B: acetonitrile and Mobile phase A: 20 mM ammonium carbonate with 0.1% ammonia hydroxide in DDW: acetonitrile (80:20, v/v). The flow rate was kept at 200 μl/min and the gradient was as follows: 0 -2min 75% of B, 17 min 12.5% of B, 17.1 min 25% of B, 19 min 25% of B, 19.1 min 75% of B, 23 min 75% of B.

*Polar metabolites data analysis*

Initial data were processed using Compound Discoverer 3.3 (Thermo Fisher Scientific) software. The detected compounds were identified by accurate mass, retention time, isotope pattern, and fragments and verified using an in-house mass spectra library. The data were normalized for Internal Standards and the weight. Polar metabolite data were processed using the web-based pipeline *MetaboAnalyst* ^2^. A comparative analysis between vPNET and sPNET was conducted using various comparison and visualization methods, including Partial Least Squares Discriminant Analysis (PLS-DA) to visualize sample separation based on metabolite profiles, Variable Importance in Projection (VIP) analysis to identify the most influential metabolites contributing to the group separation, an unsupervised hierarchical clustering heatmap to display the expression levels of metabolites across samples, indicating patterns and similarities within and between the vPNET and sPNET groups, and a Volcano plot to highlight significantly differentially expressed metabolites.

*snRNA Sequencing Analysis*

A total of 11 PNETs were subjected to single-nucleus RNA sequencing analysis. The data were processed using the Seurat^3^ package in the RStudio software environment, using the default parameters. Data quality validation and data filtering for generating the Seurat objects were based on the following parameters and thresholds: cells expressing at least 200 and no more than 2500 genes were included, only genes that were expressed in at least three single cells were included, and cells in which >5% of the genes expressed were mitochondrial genes were excluded from the downstream analysis. Overall, four samples were removed from further analysis. Data were further normalized using the *NormalizeData* function, the top 2000 highly variable genes were selected using the *FindVariableFeatures* function, and data were scaled using the *ScaleData* function. Principal component analysis (PCA) was produced using *RunPCA*, and clusters were then defined using *FindNeighbors* and *FindClusters* functions with a resolution parameter set at 0.5. Finally, uniform manifold approximation and projection (UMAP) was used for dimensionality reduction and cluster identification using the *RunUMAP* function. Cell types in the defined clusters based on known marker genes^4^ or based on the top five uniquely differentially expressed genes of each clusterThe *SCEVAN* package^5^ was employed to identify copy number alterations, commencing with a raw count matrix of scRNA data, and categorizing the cells present in the sample by segregating non-malignant cells of the tumor microenvironment from the malignant cells, while also characterizing the clonal structure of these malignant cells. The R package *SCPA*^6^ was utilized for pathway analysis of malignant cells data, this package utilizes a comprehensive gene set that includes all the pathways from the Hallmark, Reactome, and KEGG databases from MSigDB. The final integration of all the samples was conducted using the Seurat *IntegrateLayers* function, using the *CCAIntegration* method. Immune cell sub-analysis included only four samples due to the small number of immune cells in the remaining samples. The *SingleR* package^7^ was utilized for automatic cluster annotation to identify immune cell types by referencing the Database of Immune Cell Expression and for validation of cell annotation based on marker genes. Cell trajectory and pseudo-time analyses were performed using *monocle3*^8^.

*Multi-omics Analysis*

We integrated snRNA sequencing data of two vPNET and three sPNET with metabolomics data from the relevant samples to investigate the relationship between hypoxia-related genes and metabolomic profiles. A set of hypoxia-related (*VEGFA, VEGFB, FLT1, HIF1A, EPO, EPOR, EPAS1, TTR, ARNT)* and PNET-related genes (*CHGA, CHGB, INS, SYP*) were selected for a targeted analysis. We utilized the *mixOmics*^9^ package to perform PLS analysis, correlating these gene expressions with the entire metabolomics dataset. Heatmaps of the PLS results were generated to enhance data interpretability to illustrate the interactions between metabolites and hypoxia-related genes. Finally, enrichment analysis of the top 50 most variable metabolites (**Supplementary Table 3**) was conducted using *MetaboAnalyst*^2^.

*Immunofluorescence staining*

Sequential multiplex Immunofluorescence staining was performed on 4 µm formalin-fixed paraffin-embedded (FFPE) sections from four samples of vPNET and four samples of sPNET, using the Leica Bond max system (Leica Biosystems Ltd, Newcastle, UK). Sections were dewaxed and pretreated with epitope-retrieval solution (ER, Leica Biosystems Ltd, Newcastle, UK), followed by incubation with primary antibodies, diluted in commercial diluent, followed by incubation with secondary antibodies. Samples staining for synaptophysin with the mouse monoclonal anti-synaptophysin (1:100, ab8049, Abcam, Cambridge, UK) and for adenosine receptor 2B with the Goat polyclonal anti-ADORA2B (1:100, PA5-18422, Thermo Fisher, Waltham, MA, USA), following the findings in the snRNA-seq and metabolomics analyses. Nuclear staining was achieved by applying Hoechst 33342 (1:100, 62249, Thermo Fisher, Waltham, MA, USA) for five minutes. Aqua Polymount (Polysciences, Warrington, PA, USA,18606) was used to mount and cover the slides.

*Statistical methods*

For differential comparisons of metabolites expression, we performed volcano plot, based on log2 fold change, and binomial comparisons, with false-discovery rate adjustments for multiple comparisons. Similar methods were used for differential gene expression based on snRNA seq data. The cutoffs used for each analysis are reported in each corresponding figure. Partial Least Squares Discriminant Analysis (PLS-DA) was computed based on the default parameters. Variable Importance in Projection (VIP) was calculated based the PLS-DA results. For all comparisons, FDR-adjusted p value of 0.05 was set as the minimal value for determining statistical significance.

*References for methods section*

1. Malitsky S, Ziv C, Rosenwasser S, et al. Viral infection of the marine alga Emiliania huxleyi triggers lipidome remodeling and induces the production of highly saturated triacylglycerol. *New Phytologist*. 2016;210(1):88-96. doi:10.1111/nph.13852

2. Chong J, Wishart DS, Xia J. Using MetaboAnalyst 4.0 for Comprehensive and Integrative Metabolomics Data Analysis. *Curr Protoc Bioinformatics*. 2019;68(1):e86. doi:10.1002/cpbi.86

3. Hao Y, Hao S, Andersen-Nissen E, et al. Integrated analysis of multimodal single-cell data. *Cell*. 2021;184(13):3573-3587.e29. doi:10.1016/j.cell.2021.04.048

4. Elgamal RM, Kudtarkar P, Melton RL, et al. An Integrated Map of Cell Type-Specific Gene Expression in Pancreatic Islets. *Diabetes*. 2023;72(11):1719-1728. doi:10.2337/db23-0130

5. De Falco A, Caruso F, Su XD, Iavarone A, Ceccarelli M. A variational algorithm to detect the clonal copy number substructure of tumors from scRNA-seq data. *Nat Commun*. 2023;14(1):1074. doi:10.1038/s41467-023-36790-9

6. Bibby JA, Agarwal D, Freiwald T, et al. Systematic single-cell pathway analysis to characterize early T cell activation. *Cell Rep*. 2022;41(8):111697. doi:10.1016/j.celrep.2022.111697

7. Aran D, Looney AP, Liu L, et al. Reference-based analysis of lung single-cell sequencing reveals a transitional profibrotic macrophage. *Nat Immunol*. 2019;20(2):163-172. doi:10.1038/s41590-018-0276-y

8. Cao J, Spielmann M, Qiu X, et al. The single-cell transcriptional landscape of mammalian organogenesis. *Nature*. 2019;566(7745):496-502. doi:10.1038/s41586-019-0969-x

9. Rohart F, Gautier B, Singh A, Lê Cao KA. mixOmics: An R package for ’omics feature selection and multiple data integration. *PLoS Comput Biol*. 2017;13(11):e1005752. doi:10.1371/journal.pcbi.1005752
