# Supplementary material for: Single‐cell transcriptomics and metabolomic analysis reveal adenosine‐derived metabolites over‐representation in pseudohypoxic neuroendocrine tumours
Source: Clin Transl Med. 2025 Feb 4;15(2):e70159. doi: 10.1002/ctm2.70159 (PMC11791754; doi:10.1002/ctm2.70159)
Supplement: Supplementary file 3 — Supporting Information [file CTM2-15-e70159-s001.docx]

**Supplementary Table 2.** Clinical metadata and analysis summary.

| Group | Age | Sex | Grade | Stage | Metabolomics | | snRNA seq | |
| --- | --- | --- | --- | --- | --- | --- | --- | --- |
|  |  |  |  |  | 1^st^ batch | 2^nd^  batch | 1^st^ batch | 2^nd^ batch |
| VHL | 41 | F | G1 | T2N1M0 |  | X | X^a^ |  |
| Sporadic | 68 | F | G1 | T3N0M0 |  | X | X^a^ |  |
| Sporadic | 75 | M | G1 | T2N0M0 |  | X |  |  |
| Sporadic | 74 | F | G2 | T1N0M0 |  | X | X |  |
| VHL | 33 | F | G1 | T2N0M0 | X |  | X |  |
| VHL | NA | NA | NA | NA | X^b^ | X^b^ | X |  |
| VHL | NA | NA | NA | NA | X^b^ | X^b^ | X^a^ |  |
| Sporadic | 69 | M | G2 | T3N1M1 |  |  | X^b^ | X^b^ |
| Sporadic | 75 | F | G1 | T3N1M0 | X |  | X^b^ | X^b^ |
| Sporadic | 67 | F | G2 | T2N0MX | X |  |  |  |
| Sporadic | 57 | F | G2 | M1 | X |  |  |  |
| Sporadic | 44 | M | G2 | T3N1Mx | X |  |  |  |

^a^Did not pass QC threshold and was excluded from the final analysis.

^b^Samples from the same patient but of a different tissue sample.
